# The Central Role of the Concept of Person in Psychological Science

**DOI:** 10.17505/jpor.2022.24853

**Published:** 2022-12-22

**Authors:** Lars-Gunnar Lundh

**Affiliations:** Department of Psychology Lund University, Sweden

In the present issue of JPOR, two pioneers in person-oriented research, John Nesselroade and Peter Molenaar, write about a crucial difference between measurement in the behavioral sciences and measurement in the physical sciences: Whereas movements of physical objects can be accounted for by external forces, the movement of humans and other animals are governed in part also by internal events (e.g., hunger, feelings, thoughts, intentions). This makes it difficult to standardize measurement in behavioral science by controlling the conditions under which the measurements are made. Whereas external conditions of performance (such as temperature, lighting, etc.) can be rigorously controlled, internal conditions of performance (such as level of understanding, expectations, interest, competitiveness, etc.) cannot. Individuals differ in these internal conditions, and each individual also differs over time in these aspects. And if the conditions of measuring are not the same, it is difficult to know to what extent the scores obtained by different participants, or by the same participant on different occasions, are sufficiently comparable for analysis.

The inability to equate the internal conditions of different individuals, and even the same individual over time in the case of repeated measurements, creates formidable difficulties for behavioral science. As Nesselroade and Molenaar (2022) point out, a common solution to these problems in experimental research is to use devices such as random assignment, but “such ‘fixes’ are aimed at average group performances rather than individual ones” (p. 44). In other words, this is to sacrifice the understanding of the individual and to settle for an understanding in terms of significant group differences and correlations at the group level.^1^ But is it really possible to develop a mature psychological science without including a focus also on the individual person?

According to Nesselroade and Moleanaar (2022), the individual “is the primary unit of analysis for studying behavior” (p. 45). To a layman, this may sound like a truism, but this standpoint still seems to represent a minority view among researchers in psychological science, where most research is focused on statistical comparisons between groups or on correlations at the group level. On a positive note, the authors think that

“there are signs that the tide may be turning. For example, in medicine, a rapidly growing emphasis on personalized diagnoses and treatment regimens reflects a renewed emphasis on focusing on the individual. Somewhat ironically, perhaps, this emphasis on individuality has been accelerated by advances in genetics” (Nesselroade & Molenaar, 2022, p. 45).

In a broad review of person-oriented methods, Julia Moeller (2021) recently made a similar point, not only with regards to developments in personalized medicine but also in view of the increased use of personalization in education and advertisement:

“personalized approaches and studies of between-person heterogeneity have gained terrain. In education, personalized learning aspires to assess person-specific learning needs to provide each student with the tailored support that they currently need... In personalized medicine – including personalized psychiatry – the patient’s individual needs are assessed for instance by sequencing their genome to identify individually matching treatments that would not necessarily work for other patients displaying similar symptoms (e.g., Jain, 2002; Senn, 2016; 2018). In personalized advertisement, people’s individual preferences are mapped to target them with advertisement fitting their individual personality and preferences” (Moeller, 2021, p. 65).

At the same time, Moeller (2021) noted that it looks “as though psychological science lags behind” (p. 66) these other areas in terms of their interest for focusing on the individual person. Consequently, she warned for

“the risk of a credibility loss, possibly similar to the one seen during the replicability crisis (Ioannides, 2005), because we have only started to understand how many of the conclusions that we tend to draw based on between-person methods are based on a misunderstanding of what these methods can tell us and what they cannot” (Moeller, 2021, p. 53).

Similar concerns were raised by Lundh (2019), who argued that psychological science is in a crisis that

“goes deeper than being merely a replicability crisis. There are also signs of a normativity crisis, due to a social incentive system that is not conducive to scientific progress, and a validity crisis due to a variable-oriented approach that is not suitable to the scientific problems that need to be solved.” (Lundh, 2019, p. 203).

## Combining a Person-Oriented Approach With a Search for General Principles

Why has it been so difficult for psychological research to take the individual person into account? All through the history of psychological science there have been voices that have emphasized the importance of focusing on the individual, and among them many prominent ones. One of the early voices was William Stern’s. In his book on general psychology from a personalistic viewpoint (Stern, 1935, English translation 1938) he argued for a *science of the person* (“Personwissenschaft”, or “personalistik”, in German) that would include several specialized disciplines for studying the person, including “the biology, physiology, pathology and psychology of the person” (Stern, 1935).

Stern had his followers, such as Gordon Allport (1962). Later James Lamiell (1981) argued along partly similar lines (for a review of the main ideas of these thinkers, see Lundh, 2015). Rae Carlson (1971) in a much-quoted paper put the question: “Where is the person in personality research?” In the 1990s, David Magnusson and Lars R. Bergman formulated a distinction between person-oriented and variable-oriented research, while developing a person-oriented approach to human development (e.g., Bergman & Magnusson. 1997; Magnusson, 1999), followed by further developments (e.g., Bergman & Andersson, 2010). And in 2004, Molenaar published his manifesto on psychology as idiographic science, with the subtitle “Bringing the person back into scientific psychology – this time forever”.

And yet the person still seems to play a rather subordinate role in present-day psychological research, where there is much more focus on statistical differences between groups of individuals, and correlations at the group level, than on developmental processes at the level of the individual. This is also true for areas such as psychotherapy research, which one might think should be extra suitable for research methods that focus on processes of change at the level of the individual (e.g., Lundh & Falkenström, 2019).

One possible misconception here is that a focus on the individual person would somehow preclude a search for general laws applicable to persons. It is true that a main purpose in a person-oriented approach would be to identify regularities in personal functioning at the level of the individual, and to study how such regularities may differ from one individual to another, and from one context to another. This, however, in no way precludes the search also for regularities that *generalize across individual persons*, and across contexts. What characteries a person-oriented research is that it *starts from the individual* and subsequently attempts to generalize from individual cases to larger groups of people and contexts. In this manner, it differs from variable-oriented research which *starts at the group level* and conducts statistical analyses of differences between groups (e.g., experimental and control groups) and correlations between different variables. The preference for person-oriented research therefore in no way implies that we should give up attempts to formulate general principles of psychological functioning; such conclusions, however, should primarily rely on comparisons between individuals, and not on statistical averages at the group level.

In their article in the present issue, Nesselroade and Molenaar (2022) approach a partly similar question in terms of a differentiation between manifest and latent variables. Manifest variables are observable and directly measurable but may have to be measured differently in different individuals. Latent variables, on the other hand, are not directly observable, and their properties have to be inferred from manifest variables which serve as indicators of these latent variables.

Behavioral science, as they formulate it, is similar to other sciences in seeking general knowledge, and a key component in developing scientific knowledge is to search for “invariant relations”:

“the invariant relations we seek as behavioral scientists are to be found among the latent variables, and the array of manifest variables indexing those latent variables might have to be different from one individual to another (Nesselroade & Molenaar, 2022, p. 47).

Speaking about “latent variables” and “invariant relations” suggests the importance of theoretical analysis, and maybe one problem for a person-oriented approach to psychological science lies in the absence of a sufficiently well-elaborated theoretical conception of the individual person.

## Towards a Theoretical Modelling of the Person

Could it be that what is required here is a well-developed theoretical model of the person? Maybe the problem is that we still lack a coherent theoretical paradigm that puts the person at the center of the stage? What is a person exactly? Intuitively, we may all feel that we know the answer to this question. But where do we find a convincing theoretical model of the person?

Some promising theoretical work on the concept of person has been carried out by researchers such as Bickhard (e.g., 2004, 2012, 2016, 2017) and Ossorio (2006). According to Bickhard (2017), persons are the loci of psychological phenomena and are therefore at the center of what psychology should study. As Bickhard argues, a basic problem with psychological research is that it operates with naïve and confused philosophical assumptions about the importance of socalled “operational definitions”, which require theoretical terms to be defined in terms of empirical measurements. Because the concept of person cannot be operationally defined, the notion of the person tends to be ignored in psychology. Psychology is therefore in need of a reform of some of its basic assumptions about the nature of science so that it can take the person into scientific account. The important thing for science is whether persons can be theoretically modelled. As Bickhard points out, well-developed sciences such as physics do not rely on operational definitions but on *theoretical models* that have *consequences* that can be tested against empirical data. What psychology needs, in Bickhard’s view, is a theoretical model of the person with consequences that can be tested against empirical data.

Because persons represent a subcategory of living individuals, this theoretical model needs to involve an accurate conception of living systems more generally. Much of Bickhard’s (2016) focus is on living systems as self-maintaining processes that are recursively self-maintaining and self-reproducing. He refers to his theoretical approach as *interactivism* and describes it as an action-based framework that relies on dynamic system theory and emergentism. Emergentism is based on the ontological assumption that the world is constituted by processes, rather than by particles, entities or substances, and that this makes organization into a causal factor. Importantly, processes are inherently organized, and differing organization can yield different causal influences on the world.

The concept of *recursive self-maintenance* is central to Bickhard; living systems can maintain their property of being self-maintenant by shifting between different processes. This represents a form of *autonomy*. Bickhard (2016) sees autonomy as an ability to make use of environments to maintain persistence, and this an ability that comes in degrees, and that can be seen in a simple form even in bacteria. More complex forms of autonomy are seen in the ability of living individuals to learn, and to make use of self-reflective forms of self-maintenance. As Bickhard (2016) sees it, all these more complex forms of autonomy can be understood within the framework of recursive self-maintenance; “self-maintenance is itself an interactive relational process, and is thus well suited as a framework for modeling the emergence of cognitive and representational relationships.” (p. 28.) In Bickhard’s (2017) view, persons are complex “socio-cultural-linguistic” agents who develop from infancy through the life-span.

Another interesting attempt to formulate a theoretical model of the person is seen in Ossorio’s (2006) work. A starting-point for his analysis is that to be a person means to have the concept of person, as a basic form of *competence*. Ossorio sees this partly on analogy with having the capacity for language. Just as we acquire linguistic competence during our psychological development and maturation, we also acquire the competence of understanding what it means to be a person during our development. This is what accounts for the basic fact that “people are not inherently mysterious to other people” (Ossorio, 2006, p. 2), and lies at the basis of our understanding of interpersonal relations. But just as people who are competent in a language find it difficult to describe all the grammatical rules that they exercise spontaneously as part of their linguistic competence, people who have acquired the person concept and can exercise this understanding spontaneously in their interpersonal interaction with others also find it difficult to describe the details of their understanding. Analyzing the concept of person and making this understanding explicit, according to Ossorio (2006), is the task for what he refers to as “descriptive psychology”.

In contrast to Bickhard, Ossorio (2006) does not focus on the kind of physical or biological underpinnings of persons that are seen in living organisms generally. Instead he starts from a psychological perspective that defines a person as “an individual whose history is, paradigmatically, a history of Deliberate Action in a Dramaturgical Pattern” (Ossorio, 2006, p. 69). Importantly, this gives a central importance to the concept of an individual’s personal *history*. As Ossorio (2006) puts it, the “appropriate size of the unit for conceptualizing a person is not a behavior but a life history” (p. 384). This suggests a holistic perspective, which conceives of individual behaviors and experience in terms of their role in the person’s life patterns.

In his analysis of the concept of person, Ossorio uses a number of notational devices including parametric analysis. A simple example of parametric analysis from another area is the dimensional analysis of visible colors in terms of brightness, intensity and hue, where each individual color can be described in terms of its values in these three dimensions. Ossorio similarly wants to identify the parameters (dimensions) that are needed to give an unambiguous description of persons and behaviors. In terms of Ossorio’s analysis, the parameters involved in the description of behavior (intentional action) among other things involve knowing and wanting. What separates deliberate action from other varieties of intentional action in his analysis is that in deliberate action the intentional action itself is included in the values of the knowing and wanting parameters. That is, when persons engage in deliberate action they know not only about their goals and possible means for reaching these but also about their intentional action itself, and what they want is not only to reach these goals but also to engage in this particular intentional action.

Maybe the future will see an increased cross-fertilization between (1) the theoretical development of a comprehensive model of the person and (2) methodological developments that facilitate the study of developmental processes at the level of the individual person. Maybe this is what is required for “bringing the person back into scientific psychology – this time forever”, as Molenaar (2004) once formulated it.

But in the meantime we may have to face the fact that some representatives of psychological science remain convinced that person-oriented research is “not relevant”. A concrete example of this was seen in the response given to the application of the *Journal for Person-Oriented Research* for indexing in PsycINFO, the database produced by the American Psychological Association. The application was rejected, and the most interesting thing about this rejection was the motivation for it. The reason for rejection was not formulated in terms of insufficient scientific *quality*, or any other aspect of *quality*, but stated simply as “lack of relevance” (email message from Jenifer Guin, JGuinn@apa.org on September 16th, 2021).

This verdict was in stark contrast with the evaluation of the *Journal for Person-Oriented Research* from PubMed, which was based on a detailed evaluation of the journal’s scientific and other qualities and led to an acceptance of indexing in PubMed. The contrast between the positive response from PubMed and the negative one from the American Psychological Association is well in line with Moeller’s (2021, p. 66) suggestion that it looks as though psychological science “lags behind” medicine and other fields in its interest for the individual person.

***Lars-Gunnar Lundh***

*Department of Psychology*

*Lund University, Sweden*

## Footnotes

1 For another critique, see Bergman (2017) where he addresses the difficulties in interpreting single individuals’ measurements. He points out that most measures in psychology are not highly reliable and that such measures do not normally allow the researcher to make inferences about single individuals. Although moderately reliable measurements may be sufficient if the scientific question can be answered by analyses of group statistics or by a model of the data that holds for all individuals in the sample, the situation is different when the focus is on interpreting single individuals’ scores: “in contexts where individual measurements are to be interpreted, high measurement precision must be given priority and taken into account in the research design” (Bergman, 2017, p. 125).
